# Supplementary material for: Metabolomic Profile of Indonesian Betel Quids
Source: Biomolecules. 2022 Oct 13;12(10):1469. doi: 10.3390/biom12101469 (PMC9599835; doi:10.3390/biom12101469)
Supplement: Supplementary file 1 [file biomolecules-12-01469-s001.zip › biomolecules-1925276-supplementary.pdf]

Metabolomic profile of Indonesian betel quids

Pangzhen Zhang[1]*, Elizabeth Fitriana Sari[2,3]*, Michael McCullough[4], Nicola Cirillo[4]

FIGURES, TABLES AND SUPPLEMENTARY MATERIAL

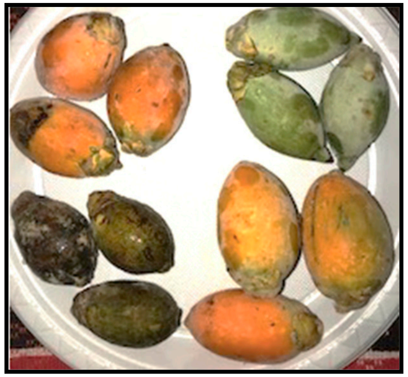

A.

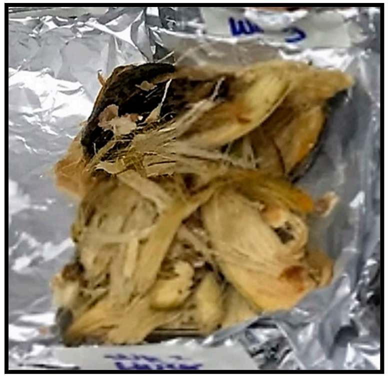

B.

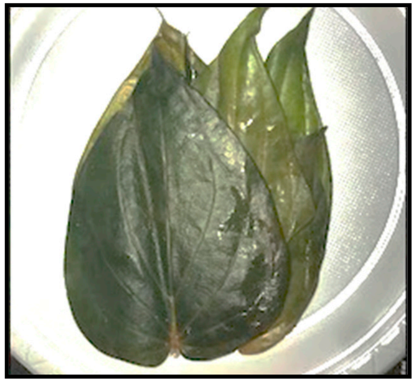

C.

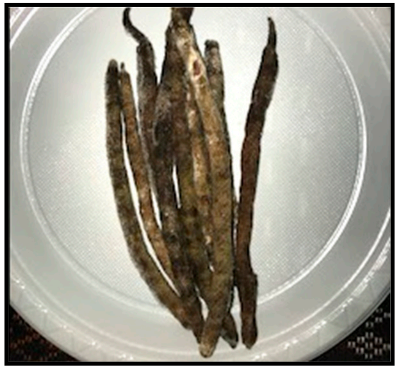

D.

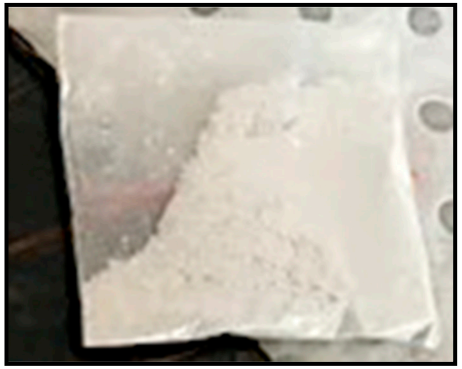

E.

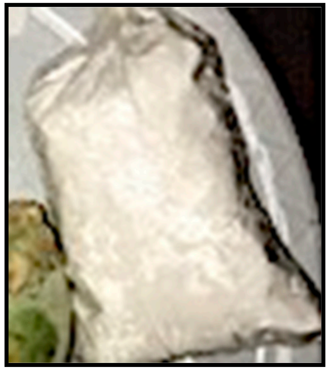

F.

Supplementary Figure S1. Areca nut (A), areca husk (B), *Piper betle* (Betel) leaf (C), Stem of *Piper betle* inflorescence (D), slaked line, powder (E) and paste (F)

Table S1. Non-targeted screening of Betel Quids ingredients using GC-MS (semi-quantified as ug 4-octanol equivalent per of sample)

Compound	Chemical	Retention time (min)	RI	RI_d[b]	Target m/z	QC m/z	BA				NS				WK				WP			
							Husk	Leaf	AN	Mix	Husk	Leaf	AN	Mix	Husk	Leaf	AN	Mix	Husk	SI	AN	Mix
2,3-Butanediol	Alcohol	2.116	806	806	45	43, 57	-	-	-	-	52.1±59.1	-	112.5±7.8	4±6.4	-	-	-	-	-	-	9.5±0.9	-
L-Lactic Acid	Acid	2.257	852	838	45	43	29.6±3.0	-	-	-	595.3±61.7	-	86.5±16.3	-	-	-	-	-	-	-	7.5±0.3	-
dl-Glyceraldehyde	Aldehyde	2.267	855	-	43	61, 60	9.8±1.0	-	-	-	85.5±8.6	1.9±15.8	8.4±0.7	-	1.6±0.3	-	-	0.4±0.1	11.6±0.8	28.4±1.3	6.6±0.7	2.3±0.2
2-Furanmethanol	Alcohol	2.333	876	875	98	81, 53, 69	2.8±0.3	-	-	-	1.1±0.2	-	-	-	-	-	-	-	6.3±0.8	-	5.9±0.5	1.5±0.1
Glycerin	Alcohol	2.71	967	-	61	43,44	33.4±1.8	27.7±1.4	-	1.3±0.1	37.4±3.7	21.7±1.8	6.6±0.8	1.9±0.4	6.6±4.3	7.2±0.7	-	-	36.1±1.7	27.6±4.7	9.8±0.6	-
D-Limonene	Monoterpene	3.125	1044	1033	68	93, 79,136	2.9±0.7	3.0±0.7	13.8±3.6	-	2.2±0.6	2±0.2	14.3±1.3	0.3±0.1	3.0±0.3	5.9±1.2	13.7±1.1	0.5±0.2	7.3±0.7	9.0±1.4	13.0±1.0	1.0±0.1
Benzeneacetaldehyde	Aldehyde	3.238	1063	1051	91	120, 65	0.9±0.1	3.5±0.2	-	-	0.6±0.1	1.3±0.1	-	-	-	3.1±0.5	-	-	3.3±0.7	0.2±0.1	1 .3±0.4	-
Linalool	Monoterpene	3.484	1103	1101	71	93, 55, 121, 136	-	7.6±0.3	-	-	-	5.7±0.2	-	-	-	6.1±0.3	-	-	-	2 .6±1.0	-	-
cis-β-Terpineol	Monoterpene	3.587	1117	1143	43	71, 93, 55	-	1.8±0.1	-	-	-	1.1±0.1	-	-	-	-	-	-	-	2.3±0.2	-	-
Phenylethyl Alcohol	Alcohol	3.663	1127	1120	91	122,65	1.5±0.2	1.2±0.1	-	-	7.7±0.9	3.4±0.1	-	-	1.3±0.2	-	-	-	0.7±0.1	-	0.9±0.1	-
Methyl nicotinate	Ester	3.852	1153	1145	109	78, 137, 51	0.4±0.2	0.4±0.1	13.2±0.7	-	-	-	6.7±0.4	-	-	-	15.4±4.1	-	2.6±0.3	-	32.7±1.8	1.0±0.1
4H-Pyran-4-one, 2,3-dihydro-3,5-dihydroxy-6-methyl-	Benzenoid	3.88	1157	1151	43	44, 101, 144	14.5±1.8	11.3±0.3	7.7±0.7	-	15.1±2.2	1.2±0.3	6.6±0.6	-	8.5±1.0	11.2±0.6	13.7±14.1	-	46.6±1.8	9.1±0.5	86.5±3.1	3.7±0.4
5-Methoxypyrrolidin-2-one	Amine	4.021	1176	-	84	60, 56, 115	2.0±0.1	25.6±2.6	-	-	-	34.9±2.0	-	-	1.0±0.2	6.2±0.2	-	-	4.0±0.3	3.0±0.2	-	-
1,2-Benzenediol	Benzenoid	4.097	1187	1197	110	64, 81, 92	2.8±0.4	2.4±0.1	-	-	-	3.1±0.9	9.2±1.5	-	2.7±0.4	6.2±1.2	-	-	-	8.1±1.9	-	-
4-Terpinenol	Monoterpene	4.181	1198	1191	71	93, 111, 154	-	3.4±0.2	-	-	-	2.1±0.1	-	-	-	-	-	-	-	6.2±0.3	-	-
Estragole	Benzenoid	4.266	1209	1206	148	147, 121, 117, 77	-	3.8±0.1	-	-	-	2.7±0.2	-	-	-	-	-	-	-	-	-	-
2,3-Dihydrobenzofuran	Benzenoid	4.332	1217	1226	120	91, 65	0.7±0.1	16.7±1.3	-	-	1.4±0.3	9.9±0.2	-	-	0.8±0.1	19.9±1.3	-	-	-	-	1.0±0.1	-

5-Hydroxymethylfurfural	Benzenoid	4.389	1224	1224	97	126,41, 69	15.1±2.3	-	-	-	0.4±0.1	-	-	-	-	-	-	-	92.2±11.2	-	18.3±1.1	0.3±0.1
1,2,3-Propanetriol, monoacetate	Ester	4.52	1237	1236	140	155, 96, 81, 53, 124	23.4±2.5	34.8±2.7	-	-	54.0±2.5	1.5±0.3	21.2±1	-	19.7±1.6	4.7±0.9	-	2.5±2.0	7 .6±1.5	5.3±1.1	82.9±32.9	-
Phenol, 4-(2-propenyl)-	Benzenoid	4.615	1241	-	43	103, 61	0.8±0.1	271.7±9.8	-	5.8±0.2	1.1±0.1	275.3±18.4	-	5.5±0.2	-	24.5±1.4	-	1.2±0.1	1.5±0.2	6.8±0.1	-	-
Safrole	Benzenoid	5.04	1251	1250	-	-	-	2.7±0.2	-	-	-	3.2±0.5	-	-	-	2.1±0.4	-	-	2.1±0.2	761.6±25.6	0.7±0.1	13.4±1.6
Decanoic acid, methyl ester	Ester	5.143	1252	1251	134	133, 107, 77	-	-	-	-	-	-	-	0.3±0.1	-	-	-	-	-	-	-	-
2-Methoxy-4-vinylphenol	Benzenoid	5.172	1279	-	96	141, 94	1.6±0.2	6.9±0.4	-	-	5.8±0.7	3.8±0.1	-	-	2.1±0.2	8.2±1.4	-	-	1.4±0.1	1.4±0.1	-	-
Phenol, 4-(2-propenyl)-, acetate	Benzenoid	5.379	1305	1291	162	104, 131, 77, 135, 51	-	147.9±12.9	-	-	-	17.8±0.7	-	-	-	1 .2±0.7	-	-	-	3.8±0.2	-	-
Eugenol	Benzenoid	5.502	1317	1324	74	87, 143, 155	-	212.3±178.2	-	-	0.7±0.1	9±0.8	-	-	0.7±0.1	4±0.1	-	0.8±0.1	1.1±0.2	487.8±18.0	-	14.1±1.6
3-Allyl-6-methoxyphenol	Benzenoid	5.634	1321	1324	150	135, 107, 77	1.1±0.1	3 9.5±9.9	-	6.2±0.4	1.4±0.1	252.8±16.2	-	5.4±0.2	1.4±0.1	356.5±17.2	-	8.6±1.3	1.7±0.2	321±274.4	-	-
alpha-Copaene	Sesquiterpene	5.794	1345	1350	134	107, 176, 77	-	9.2±0.2	-	-	-	6.8±0.7	-	-	-	7.1±0.5	-	-	-	285.2±1.0	-	3.0±0.2
Methyleugenol	Benzenoid	5.841	1359	1359	164	149, 77,131	-	2.1±0.1	-	-	-	1.7±0.1	-	-	-	1.6±0.1	-	-	-	89.2±2.8	-	1.1±0.1
1H-Cyclopropa (a)naphthalene, 1a,2,3,5,6,7,7a,7b-octahydro-1,1,7,7a-tetramethyl-, (1aR-(1a.alpha.,7.alpha.,7a.alpha.,7b.alpha.))-	Sesquiterpene	5.879	1375	1362	164	149, 103, 91,77, 55	-	14.0±0.6	-	-	-	9.5±0.8	-	-	-	8.7±0.9	-	-	-	169.1±7.2	-	1.4±0.1
Methyl 5-formyl-1H-pyrrole-3-carboxylate	Benzenoid	5.983	1394	1387	161	119, 105, 93	-	-	-	0.8±0.2	-	-	-	1.4±0.4	-	-	-	1.6±0.3	-	-	-	-
α-Bergamotene	Sesquiterpene	6.077	1399	1401	178	147, 163, 103, 107	-	8.4±0.1	-	-	-	5.0±0.5	-	-	-	1.1±0.2	-	-	-	-	-	-
alpha-gurjunene	Sesquiterpene	6.124	1403	-	161	105, 119, 91, 204	-	-	-	-	-	-	-	-	-	-	-	-	-	1 4.6±1.5	-	1.1±0.1
α-Santalene	Sesquiterpene	6.153	1414	-	122	153, 94	-	3.6±0.1	-	-	-	2.1±0.3	-	-	-	-	-	-	-	-	-	-
beta-Ylangene	Sesquiterpene	6.219	1423	1416	93	119, 41, 69, 77	-	6.8±0.3	-	-	-	3.6±0.3	-	-	-	3.2±0.1	-	-	-	23.1±0.2	-	-
(E)-β-Caryophyllene	Sesquiterpene	6.266	1428	1413	204	161, 189, 105	-	36.6±1.3	-	-	-	24.3±2.0	-	-	-	2 .1±1.0	-	-	-	146±4.0	-	1.2±0.1
cis-Muurola-3,5-diene	Sesquiterpene	6.341	1430	1424	94	93, 107, 79, 69, 121	-	9.3±0.2	-	-	-	5.4±0.4	-	-	-	4.9±0.1	-	-	-	35.1±0.6	-	-
alpha-humulene	Sesquiterpene	6.624	1437	1420	161	120, 105, 91	-	34.1±1.7	-	-	-	24.2±1.9	-	-	-	8.6±0.3	-	-	-	55.7±1.5	-	-
gamma-Amorphene	Sesquiterpene	6.756	1442	1420	93	133, 69, 79, 120	-	65.9±0.7	-	-	-	48.7±3.8	-	0.8±0.1	-	19.4±1	-	-	-	71.4±2.3	-	-
γ-Cadinene	Sesquiterpene	6.879	1449	1447	161	105, 93, 69	-	15 .5±5.0	-	2.3±0.1	-	67.9±5.1	-	1.1±0.1	-	57.8±2.9	-	0.9±0.1	-	54.7±8.0	-	4.0±0.2
2,4-Di-tert-butylphenol	Benzenoid	6.916	1477	1489	93	80, 121, 147	6.7±0.4	8.5±0.4	3.2±0.1	-	6.4±0.5	8.8±0.2	3.4±0.2	0.7±0.1	6.1±0.1	8.1±0.3	3.8±0.2	-	6.7±0.4	-	3.0±0.1	-

3-Allyl-6-methoxyphenyl acetate	Benzenoid	7.058	1490	1492	161	119, 105, 91, 204	-	259.3±9.5	-	-	-	162.6±6.8	-	0.5±0.1	-	446.7±15.8	-	1.5±0.4	-	1158.6±31.9	1.1±0.5	-
Dodecanoic acid, methyl ester	Ester	7.058	1502	1507	161	105, 91, 119, 204	-	1.3±0.1	-	15.4±0.5	-	1.6±0.2	-	15.7±2.7	-	2.1±0.1	-	-	-	5.2±0.1	-	-
Isocalamenene	Sesquiterpene	7.284	1505	1502	191	57, 206	-	2.1±0.2	-	-	-	1.6±0.2	-	-	-	1.8±0.1	-	-	-	95.0±3.2	-	-
Selina-3,7(11)-diene	Sesquiterpene	7.332	1517	-	164	149, 131, 206	-	6.4±0.1	-	-	-	5.1±0.2	-	-	-	-	-	-	-	-	-	-
Dodecanoic acid	Acid	7.407	1517	1521	74	87, 143, 171, 55	1.6±0.1	0.7±0.2	-	-	0.6±0.1	1.5±0.3	1.0±0.2	-	0.8±0.2	1.4±0.2	1.1±0.2	-	0.7±0.1	1.6±0.2	-	-
w-Amorphene	Sesquiterpene	7.407	1537	1527	159	202	-	0.8±0.2	-	-	-	-	-	-	-	0.7±0.1	-	-	-	24.3±0.7	-	-
Isovanillic acid	Acid	7.427	1541	5142	161	122, 107, 91, 204	2.4±0.8	0.4±0.1	-	-	1 .4±1.6	-	-	-	5.5±0.2	-	-	-	1 .4±0.4	-	-	-
nerolidol ?	Sesquiterpene	7.567	1547	1562	73	60, 12, 157, 200	-	1.5±0.2	-	-	-	1.2±0.2	-	-	-	0.8±0.1	-	-	-	16.9±0.2	-	-
1,3,5-Benzenetriol	Benzenoid	7.775	1547	1540	119	105, 161, 204	-	-	56.2±15.0	-	-	-	6 .7±4.9	-	-	-	55.9±9.7	-	-	-	-	-
Spathulenol	Sesquiterpene	8.001	1549	-	168	153, 97, 125	-	2.9±0.2	-	-	-	2.8±0.2	-	-	-	71.2±58.8	-	-	-	287.7±238.6	-	-
4-Allyl-1,2-diacetoxybenzene	Benzenoid	8.407	1561	1565	69	93, 107, 136	3.3±0.1	42.9±0.9	-	-	2.2±0.2	-	-	-	1.6±0.2	372.4±11.1	-	-	-	-	3.6±0.2	-
unknown sesquiterpene 1	Sesquiterpene	8.831	1578	-	126	85, 69, 97	-	-	-	-	-	2.4±0.1	-	-	-	1.5±0.3	-	-	-	2 .8±0.6	-	-
alpha.-Cadinol	Sesquiterpene	8.963	1597	1581	43	91, 119, 205	-	0.6±0.1	-	-	-	0.5±0.1	-	-	-	0.4±0.3	-	-	-	4.6±0.4	-	-
unknown sesquiterpene 2	Sesquiterpene	9.029	1628	1638	150	192, 133	-	0.7±0.1	-	-	-	1.0±0.2	-	-	-	0.4±0.1	-	-	-	1.3±1.1	-	-
unknown sesquiterpene 3	Sesquiterpene	9.397	1660	-	161	120, 105, 204	-	1.7±0.3	-	-	-	-	-	-	-	1.1±0.1	-	-	-	12.9±4	-	-
Tetradecanoic acid, methyl ester.	Ester	9.652	1669	1662	43	95, 121, 204	-	-	-	26.7±1.2	-	0.9±0.1	-	27.2±4.8	-	-	-	9.8±0.2	-	-	-	-
unknown sesquiterpene 4	Sesquiterpene	9.68	1674	-	43	79, 93, 105	-	-	-	-	-	-	-	-	-	-	-	-	-	1.6±1.4	-	-
4-((1E)-3-Hydroxy-1-propenyl)-2-methoxyphenol	Benzenoid	9.935	1702	-	109	91, 159, 220	6.1±0.3	1.2±0.1	-	-	8.6±1.1	-	-	-	1.5±0.6	0.9±0.1	-	-	4.2±0.1	0.9±0.1	-	-
Tetradecanoic acid	Acid	10.076	1719	1719	74	87, 143, 199	1.3±0.3	0.7±0.1	-	-	-	1.9±0.2	1.6±0.2	-	1±0.2	1.8±0.4	2.1±0.2	-	-	-	-	-
Tetradecanoic acid, ethyl ester	Ester	10.661	1721	-	43	91, 123, 220	8.3±0.9	-	-	-	0.9±0.1	2.8±0.3	-	-	-	-	-	-	2.8±0.2	-	-	-
Syringic acid	Benzenoid	10.794	1738	1729	137	180, 124, 73, 91	4.1±0.4	-	-	-	2.1±0.2	-	-	-	1.5±0.1	-	-	-	2.4±0.2	-	-	-
Pentadecanoic acid	Acid	11.632	1747	1751	73	60, 129, 228	0.6±0.1	-	-	-	1.3±0.3	-	-	-	1±0.1	-	-	-	-	-	-	-
Pentadecanoic acid, ethyl ester	Ester	12.236	1786	1790	88	101, 157, 256	2.4±0.2	-	-	-	1.7±0.2	-	-	-	-	-	-	-	2.2±0.2	-	-	-
Hexadecanoic acid, methyl ester	Ester	12.773	1795	-	198	183, 127, 109	0.9±0.1	1.7±0.1	-	17.9±0.6	0.8±0.1	6.3±0.6	-	16.4±2.9	-	3.5±0.3	-	6.4±0.1	1.0±0.2	3.1±0.1	-	0.8±0.2

Methyl 3-(3,5-di-tert-butyl-4-hydroxyphenyl)propionate	Benzenoid	12.896	1848	1851	73	60, 129, 242	2±0.1	–	0.8±0.2	–	1.8±0.2	2.2±0.3	0.8±0.1	–	-	-	0.9±0.1	-	-	2.1±0.1	0.7±0.1	-
n-Hexadecanoic acid	Acid	13.282	1886	1890	88	101, 157, 270	8.7±0.4	12.4±1.9	–	–	13.7±1.6	22.7±3.5	1.6±0.1	–	12.8±1.1	15.5±1.1	2.4±0.1	-	8.3±0.7	14.7±0.6	1.3±0.4	-
Hexadecanoic acid, ethyl ester	Ester	13.896	1919	1921	74	87, 143, 227, 270	39.5±3.8	3.0±0.1	–	–	22.3±2.4	12.1±0.8	–	–	2.0±1.1	1.1±0.1	-	-	89.5±4.4	2.1±0.1	-	-
Heptadecanoic acid, methyl ester	Ester	14.443	1927	1943	277	292, 219, 147	–	–	–	–	–	–	–	–	-	-	-	-	-	-	-	-
Heptadecanoic acid	Acid	14.952	1950	1951	73	60, 129, 256	–	–	–	–	0.5±0.2	–	–	–	-	-	-	-	-	-	-	-
Heptadecanoic acid, ethyl ester	Ester	15.574	1987	1991	88	101, 157, 284	1.4±0.1	–	–	–	1.1±0.2	–	–	–	-	-	-	-	3.2±0.1	-	-	-
8,11-Octadecadienoic acid, methyl ester	Ester	15.6	2020	2022	74	87, 143, 241, 284	–	0.4±0.1	–	8.6±0.3	–	1.7±0.1	–	8.5±1.6	-	0.5±0.1	-	-	1±0.2	-	-	0.6±0.1
9,12,15-Octadecatrienoic acid, methyl ester, (Z,Z,Z)-	Ester	15.678	2050	2065	73	129, 270	–	1.0±0.1	–	2.0±0.1	0.5±0.1	3.2±0.4	–	1.7±0.4	-	1.6±0.2	-	-	-	0.6±0.1	-	-
9-Octadecenoic acid, methyl ester, (E)-	Ester	15.716	2087	2089	88	101, 55, 298	–	0.7±0.1	–	9.5±0.1	0.5±0.1	2.5±0.3	–	9.1±1.5	-	1.2±0.2	0.8±0.2	3.9±0.1	0.5±0.1	1.4±0.1	-	0.3±0.1
Phytol	Diterpene	15.848	2089	-	67	81, 95, 294	–	–	–	0.7±0.1	0.7±0.1	82.4±5.1	–	0.7±0.1	-	35.2±2.6	-	-	2.8±0.3	8.1±0.3	-	-
Methyl stearate	Ester	16.121	2093	2092	79	67, 95, 108, 292	–	–	–	2.8±0.2	–	2.5±0.2	–	2.8±0.5	-	1.1±0.1	-	-	-	-	-	-
9,12-Octadecadienoic acid (Z,Z)-	Acid	16.168	2096	2084	55	69, 83, 264	3.7±0.1	3.7±0.7	–	–	7.1±1.0	7.8±2.1	–	–	3.7±0.5	3.1±0.5	-	-	7.2±0.2	9.0±0.2	-	-
9,12,15-Octadecatrienoic acid, (Z,Z,Z)-	Acid	16.282	2104	2105	71	81, 57, 123	3.8±0.3	23.7±4	–	–	7.5±0.7	19.2±5.9	–	–	3.2±0.5	23.5±1.7	-	-	7.1±0.3	6.8±0.7	-	-
Octadecanoic acid	Acid	16.659	2120	2117	74	87, 143, 298	0.4±0.4	–	–	–	0.3±0.1	–	–	–	0.2±0.1	-	-	-	-	-	-	-
Linoleic acid ethyl ester	Ester	16.697	2123	2126	67	81, 95, 55, 43	13.4±1.3	1.7±0.1	–	–	8.5±1.0	5.5±0.6	–	–	0.9±0.5	-	-	-	41.7±2.6	2.3±0.1	-	-
Ethyl Oleate	Ester	16.8	2130	2134	41	79, 67, 55, 95, 108	15.3±1.3	–	–	–	9.8±1.0	4.3±0.6	–	–	0.6±0.3	1.0±0.2	-	-	28.2±1.5	2.1±0.4	-	-
Octadecanoic acid, ethyl ester	Ester	17.254	2152	2158	43	73, 55, 60, 129, 284	5.7±0.5	1.4±0.1	–	–	3.3±0.4	5.4±0.4	–	–	-	-	-	-	6.8±0.3	0.5±0.1	-	-
Phytol, acetate	Ester	17.564	2154	2155	67	81, 95, 208	0.6±0.1	5.8±0.1	–	–	–	17.6±0.9	–	–	-	2.5±0.1	-	-	-	-	-	-
Tetradecanoic acid, 2,3-dihydroxypropyl ester	Ester	19.027	2161	2180	55	41, 69, 88, 97	–	–	–	1.9±0.2	–	–	–	2.1±0.5	-	-	-	-	-	-	-	-
Tetradecanoic acid, 2-hydroxy-1-(hydroxymethyl)ethyl ester	Ester	19.177	2188	2188	88	101, 157, 312	–	–	–	–	–	–	–	0.3±0.1	-	-	-	-	-	-	-	-
Eicosanoic acid, methyl ester	Ester	19.442	2206	2218	43	68, 123, 95, 278	–	–	–	–	–	–	–	0.3±0.1	-	-	-	-	-	-	-	-

9-Octadecenamide, (Z)-	Amine	19.941	2295	-	211	43, 57, 98, 74	4.8±2.6	6.1±0.6	-	0.4±0.1	4.8±0.3	8.6±0.5	-	-	4.6±0.3	8.0±1.0	0.9±0.2	0.3±0.1	5.6±1.2	4.4±0.6	-	-
Eicosyl acetate	Ester	20.733	2305	-	211	98, 43, 57, 74	-	0.8±0.1	-	-	-	3.0±0.2	-	-	-	0.5±0.1	-	-	-	-	-	-
Hexadecanoic acid, 2-hydroxy-1-(hydroxymethyl)ethyl ester	Ester	22.299	2321	2329	74	87, 143, 283, 326	1.2±0.1	4.8±0.3	-	0.7±0.1	0.5±0.1	5.1±0.2	-	0.7±0.1	0.9±0.1	6.2±0.3	-	-	1.9±0.3	1.8±3.1	-	-
Glyceryl monolinoleate	Ester	24.94	2352	-	59	72, 41, 126	-	2.0±0.2	-	0.9±0.1	-	2.4±0.4	-	0.9±0.2	-	1.8±0.2	-	-	1.2±0.2	3.2±0.2	-	-
β-Glyceryl monostearate	Ester	25.015	2402	2410	43	57, 97, 340	-	2.5±0.2	-	1.9±0.1	-	5.2±1.0	-	2.0±0.5	-	5.9±0.3	-	-	-	-	-	-

All compounds were semi-quantified as 4-octanol equivalent. RI, retention index experimental; RI_{db}, retention index database.
